# Effect of a Combined Exercise and Dietary Intervention on Self-Control in Obese Adolescents

**DOI:** 10.3389/fpsyg.2019.01385

**Published:** 2019-06-28

**Authors:** Ming-Qiang Xiang, Jing-Wen Liao, Jun-Hao Huang, Hai-Lin Deng, Dan Wang, Zebo Xu, Min Hu

**Affiliations:** ^1^Department of Sports and Health, Guangzhou Sport University, Guangzhou, China; ^2^Guangdong Provincial Key Laboratory of Sports and Health Promotion, Guangzhou Sport University, Guangzhou, China

**Keywords:** combined exercise and dietary intervention, obese adolescents, cognitive self-control, physical self-control, body mass index

## Abstract

**Objective:**

The aim of this study was to determine whether a combined exercise and dietary intervention improved cognitive and physical self-control and whether pre-to-post interventional changes in self-control were mediated by changes in body mass index (BMI) and maximal grip strength (MGS), in a sample of obese adolescents.

**Methods:**

Forty-four obese adolescents were randomly assigned to a combined exercise and dietary program or to a waitlist control group; the data from 36 participants (*n* = 18 for each group) were analyzed. The combined exercise and dietary program was performed over 6 weeks and was supervised by qualified trainers in a closed boot camp. The exercise consisted primarily of typical aerobic training, sports, outdoor training, yoga, and resistance training. Participants were placed on moderate dietary restriction according to individual target body weight (30 kcal/kg × target weight). The primary outcomes of this study were metrics based on cognitive and physical self-control, assessed by the Stroop task and a handgrip task, respectively. Secondary outcomes included BMI and MGS.

**Results:**

The combined exercise and dietary intervention significantly improved both cognitive and physical self-control. Similar positive effects were also found for reduced BMI and enhanced MGS. Correlation analyses showed that the reduced BMI and enhanced MGS were significantly closely associated with improved cognitive and physical self-control. The mediation analyses revealed that the pre-to-post intervention changes in BMI and MGS significantly mediated physical self-control, but did not mediate cognitive self-control.

**Conclusion:**

Our combined exercise and dietary intervention is an effective approach to improve multiple aspects of self-control, reduce BMI, and strengthen MGS among obese adolescents. These findings also suggest that reduced BMI and enhanced MGS mediate specific aspects of self-control.

## Introduction

With the adoption in recent years of high caloric intake and sedentary lifestyles, the prevalence of obesity in childhood and adolescence has steadily increased worldwide, including in China. A study by Zhang et al. (2018) showed that approximately 15.5% of Chinese children and 8.8% of adolescents were overweight or obese; moreover, the prevalence of obesity in adolescents aged 12–18 years has significantly increased between 2011 and 2015. Childhood and adolescence obesity are linked to numerous comorbidities in adulthood, such as diabetes, cardiovascular disease, and metabolic syndrome (Biro and Wien, 2010; Doyon and Schaefer, 2013; Liang et al., 2015).

Adverse obesity-related health consequences have been linked to poor self-control. Self-control involves the capacity to volitionally overcome dominant behaviors or to resist temptation in order to achieve a particular goal, and is integral to the successful navigation of daily life (Baumeister et al., 1998; Englert, 2016). For obese individuals, maintenance of long-term lower weight may require strong self-control in order to consistently reinforce positive health-behavior lifestyles. For example, Bickel et al. (2018) showed that individuals who succeed in maintaining their weight loss exhibited superior self-control compared to those of the control group. However, obesity associated with poorer self-control than in control subjects has been specifically reported across a variety of personality and behavioral measures (Elfhag and Morey, 2008; Chamberlain et al., 2015). A meta-analysis showed significant deficits in inhibitory control in obese participants compared with those in healthy-weight controls [*g* = −0.363, 95% confidence interval (CI): −0.473, −0.252] (Yang et al., 2018a). Importantly, Datar and Chung (2018) found that poor self-control in school-aged children was an important risk factor for an increase in unhealthy body mass index (BMI) during the transition to adolescence over the next 8 years. Therefore, Datar and Chung (2018) suggested that a better understanding of ways to promote self-control may be important for improving the effectiveness of obesity-prevention programs.

According to the strength model of self-control (Baumeister et al., 1998; Muraven et al., 1999), self-control is a domain-general resource. Just as a muscle gets stronger with regular exercise, self-control may be strengthened with repeated regular practice. Several interventions seeking to improve self-control in obese adolescents or adults have shown promise, including self-management training (Vinkers et al., 2014), self-administered internet-based training (Mensorio et al., 2019), executive-function training (Verbeken et al., 2013), and response inhibition to food training (Lawrence et al., 2015; Verbeken et al., 2018).

Among various self-control training protocols, exercise has been regarded as an effective intervention, which has been observed to have benefits for improving the self-control abilities among female college students (Zou et al., 2016), children (Best, 2010), elderly people (Colcombe and Kramer, 2003), and patients with schizophrenia (Li et al., 2014). The benefits of exercise have prompted researchers to adopt exercise training to improve the self-control of overweight or obese individuals. Recently, Liu et al. (2018) demonstrated that coordination-exercise intervention not only improved the physical fitness of obese adolescents and reduced their BMI, but also enhanced their cognitive inhibitory control in the domain of executive function, which was reflected by the results of a normal and food-cue-related Stroop task. Furthermore, a series of randomized controlled trials in overweight children provided evidence of exercise-induced changes in activating their brains, including in the prefrontal cortex and the anterior cingulate cortex (Davis et al., 2011; Chaddock-Heyman et al., 2013; Krafft et al., 2014), which are areas of the brain linked to self-control (Kelley et al., 2015). More importantly, improving self-control may help to enhance health-behavioral options and to overcome unhealthy choices. For example, exercise-induced improvements of cognitive performance, specifically inhibitory control, were recently demonstrated to transfer to self-control in the dietary domain on a following laboratory taste test that included high-energy foods (Lowe et al., 2016). Nonetheless, these previous studies were limited to exercise training alone for improving the self-control of obese individuals and were not combined with dietary intervention. Exercise and diet are well known as the mainstays of obesity treatment, and combined exercise and dietary interventions result in greater weight loss than dietary or exercise interventions alone (Ho et al., 2013; Johns et al., 2014). Clearly, more empirical studies are required to investigate the efficacy of combined exercise and dietary interventions for improving self-control of obese individuals.

Prior studies merely tested the cognitive aspect of self-control in obese individuals, reflected by the Stroop task. However, self-control can be subdivided into other dimensions besides cognitive control, such as physical control. Cognitive self-control refers to an individual’s concentration on thinking to complete tasks related to goals, while physical self-control refers to the ability of individuals to overcome potential impulses and to consistently complete motor tasks (Hagger et al., 2010; Berkman et al., 2012). Whether or not these two types of self-control can be improved through combined dietary and exercise interventions for obese individuals remains unknown.

Adolescence is a key transitional stage in terms of physical and mental development, during which personal lifestyle choices and behavioral patterns are established. The purpose of the present study was to assess the effects of combined exercise and dietary intervention on cognitive and physical self-control, as well as BMI and maximal grip strength (MGS), among obese adolescents. An additional purpose of the present study was to assess whether pre-to-post interventional changes in self-control were mediated by changes in BMI and MGS. The primary hypotheses were that a combined exercise and dietary interventional program would improve both cognitive and physical self-control, as well as reduce BMI and strengthen MGS. Secondary hypotheses were that reduced BMI and enhanced MGS would mediate improved cognitive and physical self-control.

## Materials and Methods

### Participants

**FIGURE 1:**
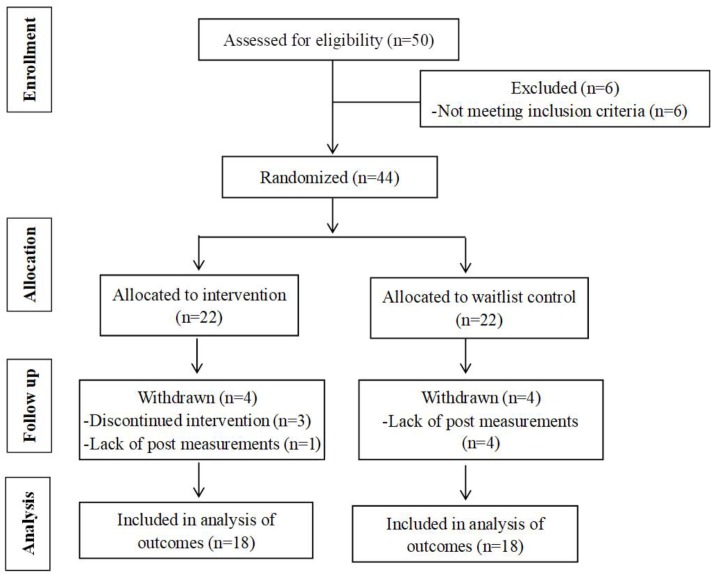
Summary of the participant-flow diagram.

A sample of 50 obese children was recruited from Shenzhen City in the Guangdong Province of China, from the beginning to the end of June 2018. Eligibility criteria included the following: (1) age range from 9 to 16 years; (2) obesity with an age- and sex-specific BMI ≥ 95th percentile (Centers for Disease Control and Prevention, 2019); (3) normal or corrected-to-normal vision without color-blindness; and (4) no self-reported history of severe cardiovascular or psychiatric disorders. According to baseline assessments, 44 qualified participants were allocated randomly—via a computer-generated algorithm—to either a combined exercise and dietary (EXD) group or a waitlist control (CON) group. Prior to the study, the parents or guardians of participants were well informed and their written consents were obtained. Thirty-six participants (*n* = 18 for each group) completed pre- and post-assessments, which were included in the statistical analysis (see flow diagram, Figure 1). In the current study, the sample size was sufficient to reveal at least a medium effect size *via* G^∗^Power analysis (Faul et al., 2007) with the following parameters: effect size: *f* = 0.25, α = 0.05, 1-β = 0.80, *r*_repeated measures_ = 0.50, ε = 1; exact sample advised: *n* = 35). This study was approved by the Guangzhou Sport University Human Experimental Ethics Board.

### Combined Exercise and Dietary Intervention

The combined exercise and dietary interventional program lasted 6 weeks (from July 2nd to August 12th of 2018) at a closed boot camp in Shenzhen, Sunstarasia.

#### Dietary Restriction

In the CON group, participants were advised not to change their energy intake. In the EXD group, subjects completed 3-day food records before the intervention. Then, a moderate dietary restriction was provided to each participant based on his or her individual target body weight (30 kcal/kg × target weight). Target body weight of each specific participant was calculated according to his or her target BMI that was based on the BMI data of Chinese children in the 50th percentile from the same age and gender (Yang et al., 2018b). Diet was nutritionally complete (20% protein, 20% fat, and 60% carbohydrate). Calorie intake ranged from 1,300 to 2,000 kcal/day based on body weight. According to an individual’s updated weight, the menu and diet were adjusted weekly during the intervention. Energy was distributed as approximately 30% at breakfast, 40% at lunch, and 30% at dinner. Mealtimes were at 7:15–7:45 a.m., 12:00 a.m.–1:00 p.m., and 5:30–6:30 p.m. every day. Registered professional dietitians determined dietary composition using Nutritionist IV software (N-Squared Computing, San Bruno, CA, United States) and prepared and monitored all meals. An example of energy intake over one day for a participant is shown in Supplementary Table S1.

#### Exercise Training

The CON group did not receive any exercise guidance and was instructed to wait until after the study before beginning any new exercise program. In the EXD group, participants performed an exercise training program for 5 h/day, 6 days/week for 6 weeks, which was conducted from 8:00 to 9:30 a.m., from 10:00 to 11:30 a.m., and from 3:00 to 5:00 p.m. every day. The exercise primarily consisted of typical aerobic training (including treadmill, dancing, rope skipping, and swimming), ball games (including basketball, badminton, and football), outdoor training, yoga, and resistance training (for more details, see Table 1). An example of a week in the training program is presented in Supplementary Table S2. The aerobic exercises included low-intensity (i.e., 2.8–4.5 METs), moderate-intensity (i.e., 4.6–6.3 METs), and high-intensity (i.e., 6.4–8.6 METs) exercise training. The intensity levels were set at approximately 50–63, 64–76, and 77–93% of the heart rate maximum (HRmax), respectively (American College of Sports Medicine, 2010). Heart rates were continuously monitored using Polar heart-rate monitors. Sport games and yoga were accommodated to one’s individual skills and rating on the perceived-exertion scale (RPE). Resistance training was performed at 40–50% maximal strength for three to four sets of 12–15 maximal repetitions. The rest period between sets and training was 60–90 s. The energy expenditure was calculated with the following equation: energy expenditure (kcal/min) = 0.0175 × weight (kg) × METs (Pinheiro Volp et al., 2011). Thus, the energy expenditure of participants ranged from 1,500 to 2,500 kcal/day during the exercise program. All the exercise programs were supervised by qualified trainers. As their exercise tolerance improved, the type of exercise program was finely adjusted and the intensity and duration of the exercise program were also progressively increased each week according to individualized data of the participants (Foster et al., 2017).

**Table 1:** Summary of the 6-week exercise training program.

Classification	Mode of exercise	Intensity	Session duration	Frequency (per week)
Endurance exercise	Rope skipping	60–75% HRmax	60–90 min	1
	Aerobic treadmill	70–90% HRmax	60–90 min	2
	Aerobic dancing	60–75% HRmax	60–90 min	1
	Swimming	60–75% HRmax	90–120 min	1
Ball games	Badminton	60–75% HRmax	90–120 min	1
	Basketball	60–75% HRmax	90–120 min	1
	Football	60–75% HRmax	90–120 min	1
Outdoor training	Hiking or climbing	40–75% HRmax	150–180 min	1
Yoga	Yoga	50–75% HRmax	60–90 min	3
Resistance training	Resistance training	40–50% maximal strength, 3–4 sets of 12–15 repetitions maximum	60–90 min	2

### Testing Procedures

All participants were required to perform measurements with two test sessions (pre- and post-training) including BMI assessment, MGS test, color word Stroop test, handgrip test, physical activity questionnaire, and trait self-control scale. Presentation of the color word Stroop test and handgrip test was counter-balanced between subjects. Each test session was conducted in a specific classroom at a training camp by well-trained graduate students. All outcome measures were performed no more than 1 week before and after the training, respectively.

### Outcome Measures

#### Self-Control Assessment

Two tasks were selected to assess self-control. A modified Stroop task was used to measure cognitive self-control and a handgrip task was used to measure physical self-control.

##### Stroop task

The modified version of the classic Stroop color–word conflict task is widely utilized to assess cognitive component of self-control (Hagger et al., 2010) and has been demonstrated to exhibit sensitivity to different exercise interventions (Chang et al., 2014; Liu et al., 2018). The stimuli were three color–word names presented in Chinese as 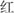 (RED), 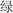 (GREEN), and 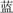 (BLUE), using E-prime 2.0 software (Psychology Software Tools, Pittsburgh, PA, United States). In the Stroop task, two types of conditions were presented. For congruent conditions, the meaning of the word matched the color ink (e.g., the word RED was presented in red ink). For incongruent conditions, the meaning of the word and color of the ink conflicted (e.g., the word RED was presented in green ink). In both conditions, the participant was instructed to identify the color of the ink. We applied Stroop interference, a specifically defined cognitive process, to elucidate the effect of combined exercise and dietary intervention on cognitive self-control. In this way, the (incongruent – congruent) contrast, which is assumed to represent Stroop interference, was calculated.

The Chinese characters used were 2 cm^2^ and each was displayed in the center of a 21-inch screen. The 96 trials in each block (48 congruent trials and 48 incongruent trials) were randomly presented. Each trial was started with the display of a white fixation point in the center of a black monitor for 500 ms, followed by a blank screen for an interval of either 300 or 500 ms (randomly selected) to avoid the participant predicting the timing of the subsequent stimulus word (Chang et al., 2014). The stimulus word was then displayed on the screen for 200 ms, which was followed by a blank screen for 2,300 ms or until the response was given.

A standard computer keyboard with “J,” “K,” and “L” buttons representing red, green, and blue, respectively, was used as the response panel. The subjects were asked to respond to the color–word by pressing a button that matched the color of ink of the word as quickly as possible with minimal error, and the reaction time and accuracy were recorded by E-prime 2.0 software. The experiment consisted of three blocks of trials, separated by 2 min of rest between each block. Twenty-four practice trials were conducted prior to the beginning of the experiment. The Stroop test lasted approximately 15 min, including practice.

##### Handgrip task

The handgrip task has been widely applied in self-control studies as a method for measuring physical self-control (Hagger et al., 2010; Tong et al., 2016). In the present study, handgrip performance was represented by the length of time (seconds) that participants were able to hold 50% of MGS on an isometric handgrip dynamometer (SAEHAN DHD-3, Saehan Corporation, South Korea) that was connected to a graphical computer interface (G-STAR software, Saehan Corporation, South Korea). To compare individual differences in strength, a relative percentage of MGS was used rather than absolute force.

Prior to the endurance trial, participants performed twice on the dynamometer with their dominant hand, with the two trials separated by 1 min of rest. The largest MGS value obtained was then halved to determine the 50% MGS target value for the endurance trials. For the endurance contraction, participants were asked to squeeze the handgrip dynamometer that included concurrent visual feedback in the form of real-time force tracing on a computer monitor. The target force level (50% MGS) was shown as a red static line on the screen. Participants were required to maintain their handgrip squeezing for as long as possible to maintain the force tracing line at, or slightly above, the target line. The trial terminated when the line tracing fell below the target-force value for longer than 2 s or when participants voluntarily gave up gripping the dynamometer. The number of seconds that the participants maintained an isometric handgrip squeeze at ≥ 50% MGS was recorded by G-STAR software and was assessed as the physical self-control performance.

#### Body Mass Index

Body mass index was carried out according to the standards of the Centers for Disease Control and Prevention (2019) and is a measure that provides BMI and the corresponding BMI-for-age percentile based on the growth charts for children and teens (ages 2–19 years).

#### Maximal Grip Strength

Maximal grip strength was measured in the dominant hand using an isometric handgrip dynamometer (SAEHAN DHD-3, Saehan Corporation, South Korea). The participants were instructed to squeeze the handle of the dynamometer maximally twice, separated by 1 min of rest. The larger value of the two measurements was recorded for purposes of analysis.

#### Physical Activity

Physical activity level was evaluated using the short form of the International Physical Activity Questionnaire (IPAQ-SF), which was developed as a global surveillance tool for physical activity (Bauman et al., 2009). Participants reported the frequency and duration of vigorous and moderate physical activities, as well as walking and sedentary activity. For each type of activity, the IPAQ-SF data were converted to a metabolic equivalent (MET min/week). The MET score weights each type of activity by energy expenditure, using 8.0 METs for vigorous activity, 4.0 METs for moderate activity, 3.3 METs for walking, and 1.0 MET for sitting^1^. Our study showed that Cronbach’s α for the IPAQ-SF was 0.310, which was similar to that of a prior study (Meeus et al., 2011).

#### Trait Self-Control

Trait self-control was assessed using the China short form of the trait self-control scale (Tan, 2008), which was developed by Tangney et al. (2004) to measure individual differences in self-control. Our study showed that Cronbach’s α for the Chinese version of the trait self-control was 0.79.

### Statistical Analysis

Independent *t*-tests were used for general profile comparisons between the EXD and CON groups in terms of demographic variables, trait self-control, and IPAQ. To evaluate the effects of combined dietary and exercise on self-control, BMI, as well as MGS, we used a mixed-design analysis of variance (ANOVA) between-subjects (group: EXD vs. CON) and within-subjects on a repeated measure (session: pre-test vs. post-test). Independent *t*-tests were also used to test for differences in pre- to post-interventional changes in self-control, BMI, and MGS between the EXD and CON groups. Bivariate correlations were applied to test the relationships between changes in cognitive self-control, physical self-control, BMI, and MGS. To further explore these relationships, we further performed mediation analyses using PROCESS software for SPSS (Hayes, 2013), based on 5,000 resamples and bias-corrected bootstrapped 95% CI. The separate mediation model postulates that the exercise and dietary intervention (predictor) would predict changed BMI and MGS (mediators), which, in turn, would predict enhanced physical and cognitive self-control (outcomes) (Fairchild and MacKinnon, 2014). The mediation model was significant if the estimated 95% CI for the indirect effect from the bootstrap test did not overlap with zero.

## Results

### Participant Demographics

Table 2 summarizes the basic descriptive characteristics for the EXD and CON groups. Independent *t*-tests indicated no significant differences between the two groups for the demographic variables of their age, height, weight, BMI, and trait self-control (*p* = 0.081–0.653). However, there was significant difference between the two groups in their MGS, with participants in the CON group showing greater MGS than those in the EXD group [*t*(34) = 2.199, *p* < 0.05]. According to the IPAQ scores, the two groups did not differ in their pre-training level of physical activity [*t*(34) = 0.499, *p* = 0.621]; however, following the intervention, participants in the EXD group scored much higher than those in the CON group [*t*(34) = 5.285, *p* < 0.001], indicating that the CON group did not take part in as much physical activity as the EXD group during the intervention.

**Table 2:** Baseline demographic characteristics of participants (mean ± SD).

Variables	CON group (*n* = 18)	EXD group (*n* = 18)
Age (years)	13.28 ± 0.83	12.50 ± 1.92
Males/females (%)	11/7 (61.11%)	9/9 (50%)
Height (cm)	164.67 ± 7.43	159.72 ± 8.98
Weight (kg)	78.58 ± 8.57	76.42 ± 18.25
BMI (kg/m^2^)	29.09 ± 2.66	29.57 ± 4.25
MGS (kg)	27.58 ± 9.21	21.76 ± 6.44^∗^
Trait self-control	3.22 ± 0.45	3.43 ± 0.52
IPAQ (METs min/week)		
Pre-training	4416.48 ± 4471.61	5100.58 ± 3728.50
Post-training	4673.44 ± 4376.30	16,785.78 ± 8680.75^∗∗∗^

*BMI, body mass index; CON, control; EXD, combined exercise and dietary; MGS, maximal grip strength; SD, standard deviation. ^∗^p < 0.05, ^∗∗∗^p < 0.001.*

### Cognitive Self-Control

Table 3 presents Stroop-task performance differences pre- and post-testing for the EXD and CON groups.

**Table 3:** Group differences across time for Stroop task, handgrip task, BMI, and MGS (mean ± SD).

Variable	CON group (*n* = 18)		EXD group (*n* = 18)	
	Pre-test	Post-test	Pre-test	Post-test
Stroop task				
Congruent (ms)	450.53 ± 119.48	490.10 ± 91.81	457.78 ± 138.44	460.48 ± 113.23
Incongruent (ms)	574.76 ± 139.78	586.56 ± 105.15	579.94 ± 146.06	507.06 ± 120.89
Stroop interference (ms)	124.23 ± 51.79	96.46 ± 44.21	122.16 ± 83.07	46.58 ± 38.89
Handgrip task				
Endurance contraction (s)	27.46 ± 15.25	28.22 ± 14.50	23.77 ± 14.99	33.43 ± 15.35
BMI (kg/m^2^)	29.09 ± 2.66	29.12 ± 2.67	29.57 ± 4.25	25.90 ± 3.79
MGS (kg)	27.58 ± 9.21	27.94 ± 9.25	21.76 ± 6.44	24.13 ± 7.09

*BMI, body mass index; MGS, maximal grip strength; SD, standard deviation.*

**FIGURE 2:**
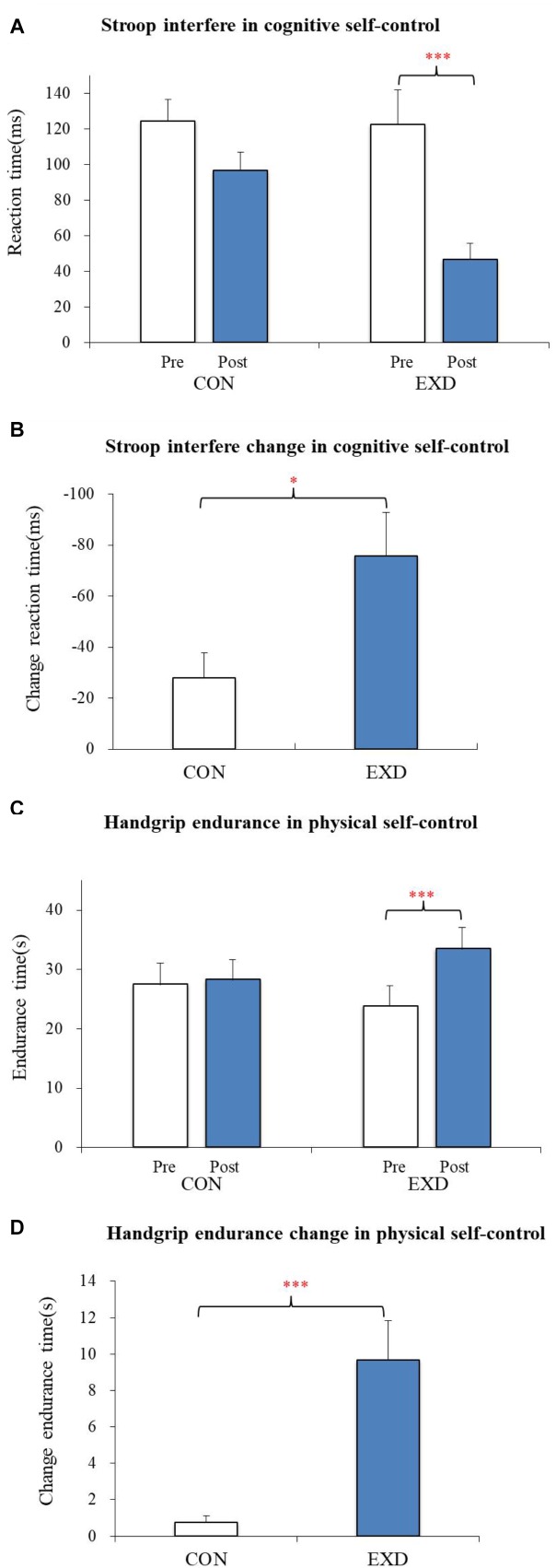
**(A)** The mean difference in reaction times between incongruent and congruent conditions, indicating Stroop interference [i.e., (incongruent – congruent)] for the waitlist control group (CON) and combined exercise and dietary group (EXD). **(B)** Stroop-interference change [i.e., (post–pre)] in reaction time for Con and EXD groups. **(C)** Mean difference in handgrip-endurance times for CON and EXD groups. **(D)** Change in handgrip-endurance time [i.e., (post–pre)] for CON and EXD groups. All data are presented as the mean ± standard error. ^∗^*p* < 0.05, ^∗∗∗^*p* < 0.001.

Results of the three-way mixed (group: EXD vs. CON) × (session: pre-test vs. post-test) × 2 (Stroop task: congruent vs. incongruent) ANOVA revealed significant main effects for Stroop condition [*F*(1,34) = 143.834, *p* < 0.001, partial η^2^ = 0.809], indicating that Stroop interference could be generally observed between the congruent and the incongruent tasks. Then, we performed a two-way mixed (group: EXD vs. CON) × (session: pre-test vs. post-test) ANOVA for Stroop interference, which is claimed by many to be one of the indicators of cognitive self-control in healthy children and young adults (Bub et al., 2006). The results showed no significant main effect by group [*F*(1,34) = 2.569, *p* = 0.119, partial η^2^ = 0.070]. There was a significant main effect by session [*F*(1,34) = 26.997, *p* < 0.001, partial η^2^ = 0.443], and the interaction between group and session was also statistically significant [*F*(1,34) = 5.777, *p* = 0.022, partial η^2^ = 0.145]. Simple-effect analyses revealed no statistical significant difference for Stroop interference in the pre-test between the EXD and CON groups [*F*(1,34) = 0.008, *p* = 0.929, partial η^2^ = 0.000]. A significantly reduced Stroop interference was observed in the EXD group [*F*(1,34) = 12.917, *p* = 0.001, partial η^2^ = 0.275], whereas the CON group showed a marginally significant change [*F*(1,34) = 3.898, *p* = 0.057, partial η^2^ = 0.103; Table 3 and Figure 2A]. In addition, the Stroop-interference-change scores were significantly reduced following exercise and dietary intervention, compared with those of control conditions [*t*(34) = 2.404, *p* = 0.022, *d* = 0.825; Figure 2B]. Together, these results indicate that exercise and dietary training may have enhanced cognitive self-control.

### Physical Self-Control

A two-way mixed (group: EXD vs. CON) × (session: pre-test, post-test) ANOVA was applied to endurance time in the handgrip task. Although no significant main effect was seen by group [*F*(1,34) = 0.024, *p* = 0.877, partial η^2^ = 0.001], a significant main effect by session was revealed [*F*(1,34) = 22.363, *p* < 0.001, partial η^2^ = 0.397], and the interaction between group and session was also statistically significant [*F*(1,34) = 16.270, *p* < 0.001, partial η^2^ = 0.324]. Simple effect analyses revealed significantly improved endurance time under the EXD group [*F*(1,34) = 38.391, *p* < 0.001, partial η^2^ = 0.530], whereas the CON group showed little to no change in endurance [*F*(1,34) = 0.2421, *p* = 0.626, partial η^2^ = 0.007] (Table 3 and Figure 2C). Endurance-time change was significantly greater in the EXD group compared with that of the CON group [*t*(34) = 4.034, *p* < 0.001, *d* = 1.384; Figure 2D], indicating that exercise and dietary training may have improved physical self-control.

### Body Mass Index

A two-way mixed ANOVA and simple effect analysis revealed that BMI was significantly lower post-test compared with pre-test for the EXD group [*F*(1,34) = 203.167, *p* < 0.001, partial η^2^ = 0.857]; however, no significant decrease in BMI was found in the CON group [*F*(1,34) = 0.011, *p* = 0.920, partial η^2^ = 0.001]. The reduction in BMI was significantly greater in the EXD group compared with that of the CON group [*t*(34) = 10.151, *p* < 0.001, *d* = 3.384], indicating that exercise and dietary training may have reduced BMI.

### Maximal Grip Strength

Two-way mixed ANOVA analyses revealed that the interaction between group and session was statistically significant [*F*(1,34) = 21.734, *p* < 0.001, partial η^2^ = 0.390]. Simple-effect analyses revealed a significantly improved MGS performance in the EXD group [*F*(1,34) = 60.313, *p* < 0.001, partial η^2^ = 0.639], whereas the CON group showed little to no change [*F*(1,34) = 1.376, *p* = 0.249, partial η^2^ = 0.039]. The improvement in MGS was significantly greater in the EXD group compared with that in the CON group [*t*(34) = 4.898, *p* < 0.001, *d* = 1.68], suggesting that exercise and dietary training may have enhanced MGS.

### Bivariate Correlation

Body mass index reduction was significantly associated with indices of Stroop-interference change (*r* = 0.361, *p* < 0.05) and handgrip-endurance change (*r* = 0.659, *p* < 0.01), with greater pre-to-post decreases in BMI correlating with greater positive change in cognitive and physical self-control performance. MGS enhancement was also significantly linked with indices of Stroop-interference change (*r* = 0.340, *p* < 0.05) and handgrip-endurance change (*r* = 0.773, *p* < 0.01), with greater pre-to-post enhancement in MGS correlating with greater positive change in cognitive and physical self-control performance. Stroop-interference change was not significantly associated with handgrip-endurance change (*r* = 0.129, *p* > 0.05), indicating that change in cognitive self-control was not linked to change in physical self-control (Table 4).

**Table 4:** Bivariate-correlation-matrix difference scores for Stroop interference, handgrip endurance, BMI, and MGS.

Variables	1	2	3	4
1 Stroop interference reduced	1			
2 Handgrip endurance improved	0.129	1		
3 BMI reduced	0.361^∗^	0.659^∗∗^	1	
4 MGS enhanced	0.340^∗^	0.773^∗∗^	0.713^∗∗^	1

*BMI, body mass index; MGS, maximal grip strength. ^∗^p < 0.05, ^∗∗^p < 0.01.*

### Mediation Analyses

The bias-corrected bootstrap method was used to test the mediation model. The results (Table 5) showed that the indirect effect of the EXD intervention improving self-control performance through changes in BMI or MGS was significant with respect to physical self-control [β_BMI_ = −0.580, and 95% CI (−1.057, −0.052), β_MGS_ = 0.447, and 95% CI (0.224, 0.705), excluding zero] but not significant for cognitive self-control [β_BMI_ = −0.105, and 95% CI (−0.573, 1.044), β_MGS_ = 0.104, and 95% CI (−0.269, 0.543), including zero]. These results indicated that BMI and MGS significantly mediated the relationship between exercise and dietary intervention and physical self-control, but did not mediate the relationship between exercise and dietary intervention and cognitive self-control.

**Table 5:** Bootstrapping indirect effect and 95% CI for the mediation model.

Indirect path	β	SE	95% CI	
			Lower	Upper
EXD intervention→BMI→cognitive self-control	−0.105	0.407	−0.573	1.044
EXD intervention→MGS→cognitive self-control	0.104	0.204	−0.269	0.543
EXD intervention→BMI→physical self-control	−0.580^a^	0.247	−1.057	−0.052
EXD intervention→MGS→physical self-control	0.447^a^	0.123	0.224	0.705

*Bootstrap sample size = 5000. β, standardized estimate of indirect effect; SE, standard error; CI, confidence interval. ^a^95% CI does not overlap with zero.*

## Discussion

In the present study, we tested whether a 6-week combined exercise and dietary intervention improved both cognitive and physical self-control, as well as reduced BMI and enhanced MGS in a sample of obese adolescents. A secondary aim was to determine whether reduced BMI and enhanced MGS outcomes mediated intervention-related improvements in self-control. Compared to the waitlist control group, the combined exercise and dietary interventional program improved both cognitive and physical self-control, reduced BMI, and enhanced MGS. Therefore, the primary hypothesis was supported. However, the pre-to-post interventional changes in BMI and MGS significantly mediated improved physical self-control but not cognitive self-control. The second hypothesis is only partially supported, suggesting that mediational effects of decreased BMI and enhanced MGS depend on the aspect of self-control.

### Beneficial Effect of Combined Exercise and Dietary Intervention

The primary findings of our study were that obese adolescents who received a combined exercise and dietary intervention showed a greater improvement in both cognitive and physical self-control compared with that of a control group, suggesting that a general improvement in self-control occurs following a 6-week combined exercise and dietary intervention. The strength model of self-control postulates that all types of self-control share the “domain general” limited resource or strength. Moreover, improvements in self-control gained within one domain may transfer to another domain, and the shared biological substrate of the right inferior frontal gyrus may play a pivotal role in this process (Baumeister et al., 1998; Berkman et al., 2012). Our results supported that improvement gained from a combined exercise and dietary intervention may transfer to improved self-control in cognitive and physical domains. On closer inspection, the effect size of change in cognitive self-control pre-to-post testing (Cohen’s *d* = 0.825) between the EXD group and CON group was slightly larger than the effect size of the change in cognitive-function performance (Cohen’s *d* = 0.56–0.60) by Liu et al. (2018)—which used coordination exercise intervention for obese adolescents—but was within the range of effect size reported in a meta-analysis by Sibley and Etnier (2003) (Cohen’s *d* = 0.00–1.49). The larger improvement of effect size in our present study might be linked to the nature of the intervention. Prior studies involved exercise intervention alone (Liu et al., 2018); however, we combined exercise and dietary intervention in a closed boot camp that was strictly monitored for the duration of the program by nutritionists and exercise trainers. Moreover, the effect size for change in physical self-control was observed to be of greater magnitude than that of the change in cognitive self-control (Cohen’s *d* = 1.384 vs. 0.825) in our intervention. To our knowledge, this is the first study in which positive effects of combined exercise and dietary intervention on physical self-control in obese adolescents have been described. One reason why improvement in physical self-control may have been greater is that our exercise approach involved a variety of exercises, such as aerobic training, ball games, outdoor training, yoga, and resistance training, which may have benefited physical self-control more than other types of self-control.

Another finding in the present study was a positive effect from the combined exercise and dietary intervention in reducing BMI, which is similar to a finding from a meta-analysis by Kelley et al. (2014) that examined the effects of exercise (strength, aerobic, or both) on overweight and obese children and adolescents (effect size = 0.47). However, our exercise program involved moderate exercise, high-intensity interval exercise, and resistance training with dietary control, and collectively lead to a large magnitude of BMI reduction (Cohen’s *d* = 3.29). Similar beneficial effects were also found for enhancement of MGS (Cohen’s *d* = 1.68). These greater changes may be associated with various aspects of the exercise intervention, such as aerobic training, ball games, outdoor training, yoga, and resistance training, which provided obese adolescents with new and stimulating experiences that many may have considered fun and motivating. In addition, given that this was a closed boot camp, many participants may have had higher internal or external motivation to shape and improve physical fitness. Taken together, this combined exercise and dietary intervention may be an effective training program for improvements in self-control and for weight control among obese adolescents.

### Mediational Effect of Body Mass Index and Maximal Grip Strength

Based on the observation of a positive effect on cognitive and physical self-control, BMI, and MGS from our combined exercise and dietary intervention, our next step was to search for relationships between reduced BMI, enhanced MGS, and improved self-control. We observed in our study that the reduced BMI was significantly associated with improved cognitive self-control (*r* = 0.361, *p* < 0.05). This finding was consistent with Xu et al. (2017), who found that improved cognitive self-control was positively correlated with weight loss in a sample of obese adolescents and young adults. Similarly, Pauli-Pott et al. (2010) found that cognitive self-control, measured by performance on go/no-go tasks, was also significantly associated with success in weight loss. Differing from previous study findings, our results demonstrated that decreased BMI was also significantly associated with improved physical self-control (*r* = 0.659, *p* < 0.01) as well as cognitive self-control. Importantly, we also observed that enhanced MGS was significantly associated with improved cognitive self-control (*r* = 0.340, *p* < 0.05) and physical self-control (*r* = 0.773, *p* < 0.01). Therefore, our findings more comprehensively revealed the relationships between decreased BMI, enhanced MGS, and the different aspects of improved self-control.

A novelty of our study was our demonstration that reduced BMI and enhanced MGS mediated intervention-related improvements in physical self-control but not cognitive self-control, indicating that the mediational effect of decreased BMI and enhanced MGS depended on the aspect of self-control. Liu et al. (2018) showed that BMI did not significantly mediate improvement of cognitive self-control performance following a 12-week program of coordination-exercise intervention. These findings were in line with our study, and plausible mediators that explain the exercise and cognition self-control relationship may not only include changes in cardiorespiratory fitness and cerebral blood flow, but may also involve brain neurotransmitters and neurotrophic factors implicated in neuronal proliferation and survival (Russo et al., 2017). We demonstrated that decreased BMI and enhanced MGS significantly mediated enhanced physical self-control performance. One reason for this inconsistency in mediational effects may be the existence of different neural mechanisms for improving self-control. Similar brain regions have been demonstrated as potential neural substrates of self-control, including dorsolateral parts of the frontal lobes, the dorsal premotor cortex, medial premotor regions, and parts of the anterior insula/frontal operculum (Langner et al., 2018); however, Kelley et al. (2015) argued that self-control includes several executive functions, each of which may have a neural signature that differs depending on specific task demands, and any one test of self-control may only influence one piece of a larger control system. Based on the results observed in this present study and those from previous neuroscience studies, we speculate that reduced BMI and enhanced MGS are more associated with the physical self-control neural signature to the extent that they mediated performance on the handgrip task. Clearly, more research is necessary to further explore whether changes in key fitness, physiological, or neural variables serve to mediate training-related improvements in self-control.

### Strengths and Limitations

There are several notable strengths to our study. First, the combined exercise and dietary intervention was conducted in a closed boot camp that could be well supervised by nutritionists and exercise trainers. Second, because cognitive and physical self-control were both measured in this study, our findings could comprehensively reveal the beneficial effects of combined exercise and dietary intervention on different aspects of self-control. Finally, we tested the mediation effect of BMI and MGS, which could contribute to understanding of how the combined exercise and dietary intervention improves self-control.

At the same time, several limitations of our study should be acknowledged. First, due to the time and resources available to complete the training program, the sample size was small. It is possible that the training was not sufficiently powered for the mediational analyses. Future studies will require sufficient sample sizes and the use of appropriate methods to investigate potential mediators of self-control based on exercise and dietary interventions. Second, our relatively short intervention (6 weeks) did not have multiple measurement points or a follow-up period. This limits the ability to reflect on clinically meaningful changes in self-control, BMI, and MGS, and whether the short-term benefits of our intervention were sustainable is unclear. Finally, we did not directly test the neural mechanisms underlying the effect of exercise and dietary intervention on cognitive and physical self-control. Future research using functional magnetic resonance imaging and/or electroencephalography will be necessary to elucidate any underlying neural mechanisms.

### Implications

Our results have several practical implications. For obese adolescents, successful maintenance of long-term lower weight requires strong self-control (Bickel et al., 2018). Although a large number of effective interventions seeking to improve self-control have been demonstrated, exercise and diet are feasible to implement in school practice as the mainstays of obesity treatment. In fact, China’s government has enacted a “National Teenagers’ Sunny Sports Program” with the goal of having students complete 1 h of exercise every day to promote physical activity (Zhang et al., 2012). In addition, there has been a growing concern about implementation of a school-based nutritional promotional program in China (Wang and Stewart, 2013). We speculate that such programs are not only useful in the field of physical fitness and preventing obesity, but also in improving self-control among obese adolescents. More importantly, improved self-control could benefit obese individuals to become more efficient in daily life when facing self-regulatory struggles such as overcoming impulses, controlling excessive eating, and breaking bad habits. Our study showed that combined exercise and dietary intervention is an effective approach for improving multiple aspects of self-control, reducing BMI, and enhancing MGS among obese adolescents in a closed camp. However, whether this benefit could extend to school-based interventions remains unclear. Thus, future research studies will be needed to explore the effect of school-based exercise and healthy-diet interventions on multiple aspects of self-control among obese adolescents.

## Conclusion

A combined exercise and dietary intervention program was effective at improving both cognitive and physical self-control, reducing BMI, and enhancing MGS among obese adolescents. The pre-to-post interventional changes in BMI and MGS significantly mediated improved physical self-control but not cognitive self-control, suggesting that the mediational effects of decreased BMI and enhanced MGS depend on the aspect of self-control. The present work extends the pediatric study on obesity, self-control, and combined exercise and dietary intervention, particularly during the critical developmental period of adolescence, and establishes the need for evidence-based public health interventions for overweight and obese adolescents.

## Data Availability

All datasets generated for this study are included in the manuscript and/or the Supplementary Files.

## Ethics Statement

The study was carried out in accordance with the recommendations from the Ethic Committee of Guangzhou Sport University with written informed consent from all participants. All participants gave written informed consent in accordance with the Declaration of Helsinki. The protocol was approved by the Ethic Committee of Guangzhou Sport University.

## Author Contributions

MH, M-QX, J-WL, and J-HH contributed to the conception and design of the study. H-LD, DW, and ZX organized the database. M-QX and ZX analyzed the data. M-QX wrote the first draft of the manuscript. MH, J-WL, and J-HH contributed to the manuscript revision, read, and approved the final version of the manuscript for submission.

## Conflict of Interest Statement

The authors declare that the research was conducted in the absence of any commercial or financial relationships that could be construed as a potential conflict of interest.

## Footnotes

1 www.ipaq.ki.se
