# Supplementary figures and images for: Effect of a Combined Exercise and Dietary Intervention on Self-Control in Obese Adolescents
Source: Front Psychol. 2019 Jun 28;10:1385. doi: 10.3389/fpsyg.2019.01385 (PMC6610291; doi:10.3389/fpsyg.2019.01385)

**Supplementary material**


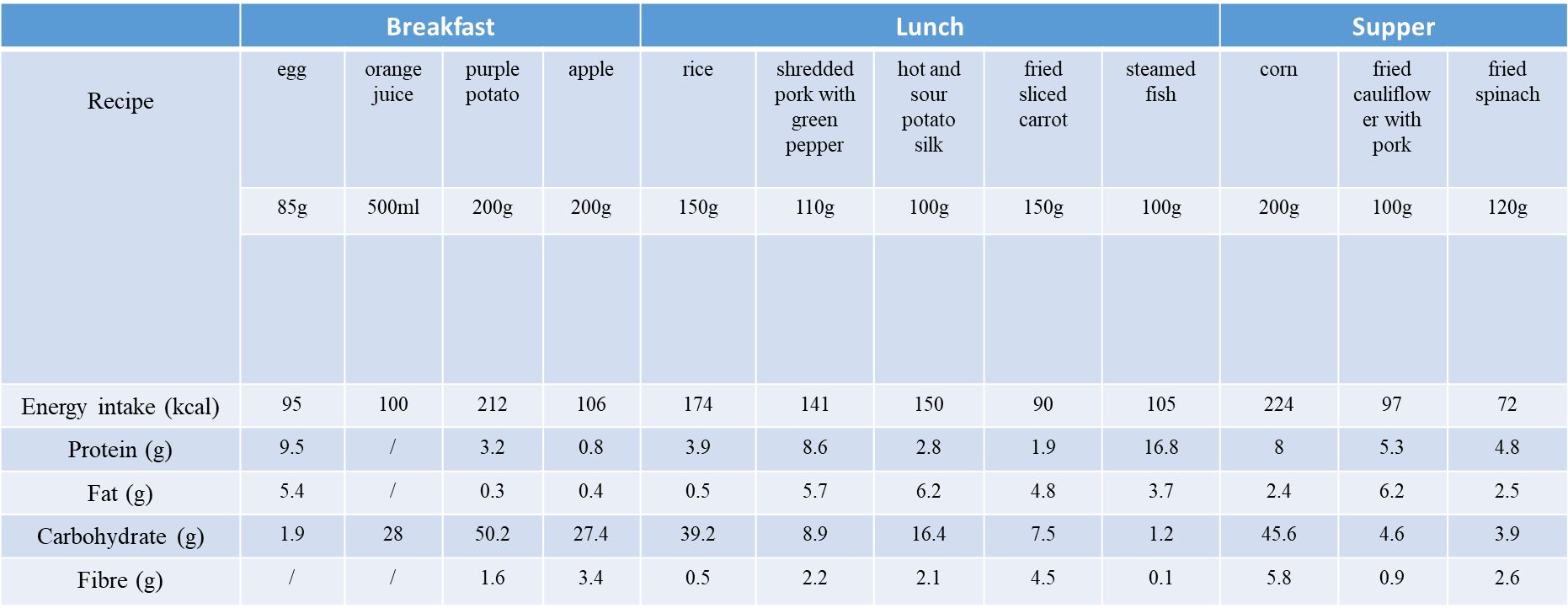

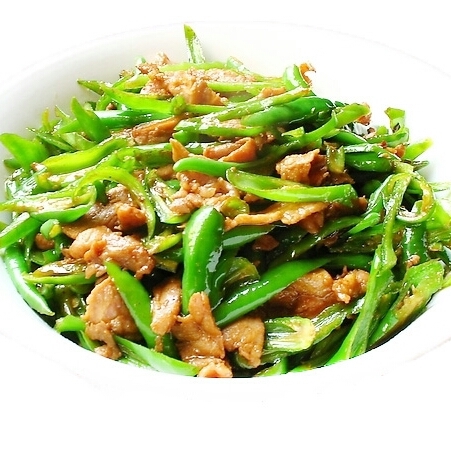

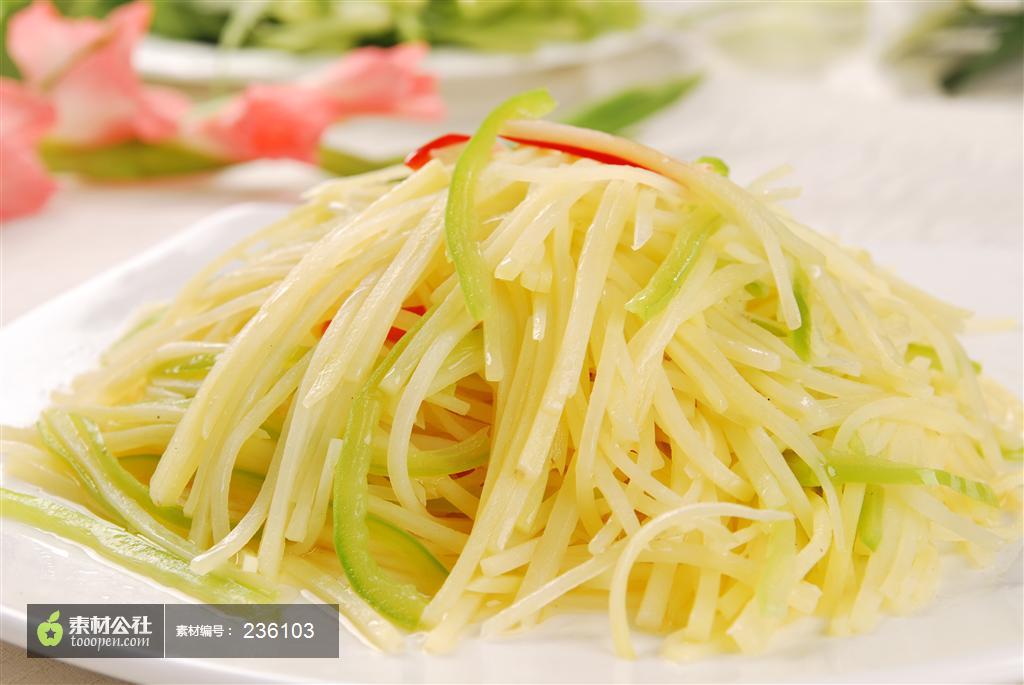

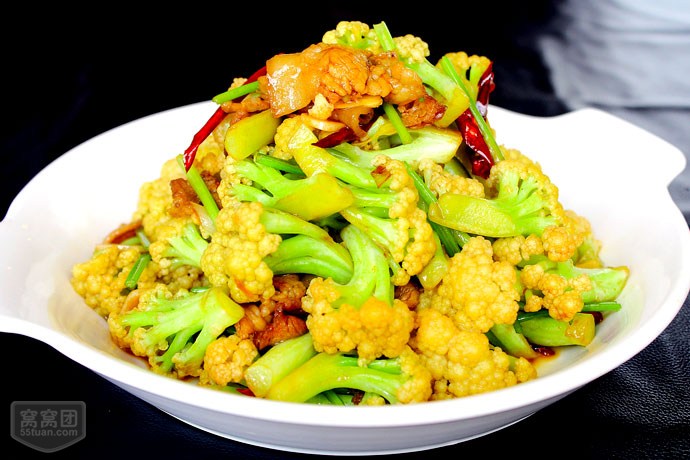

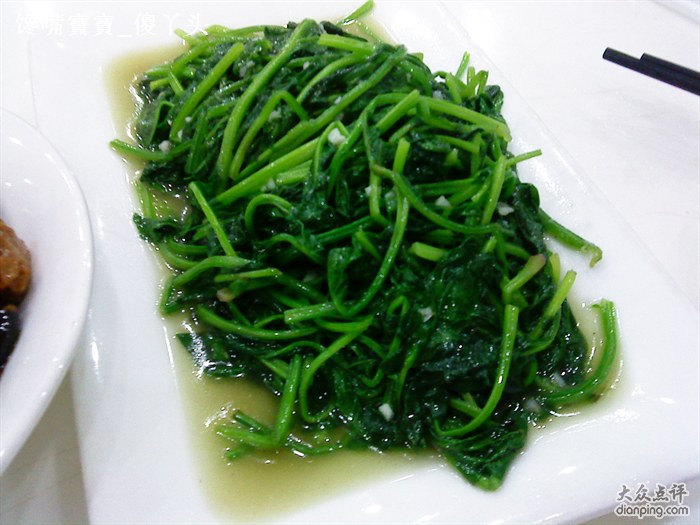

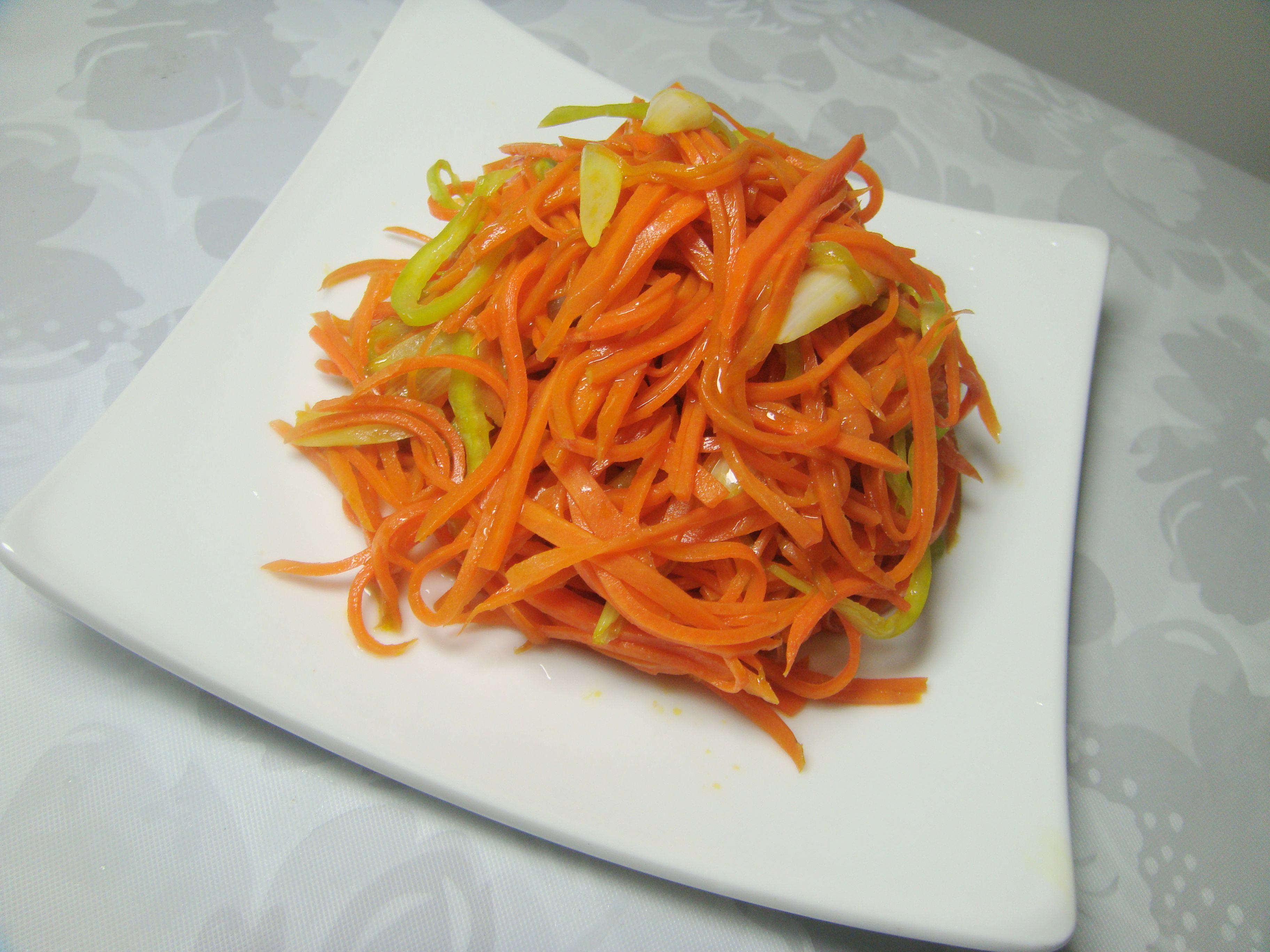

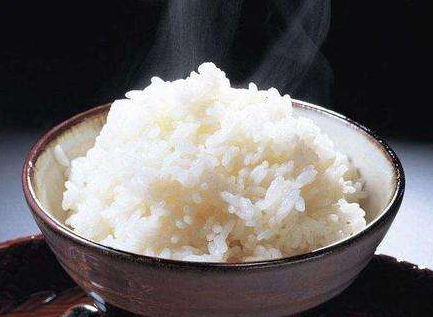

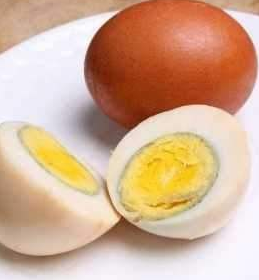

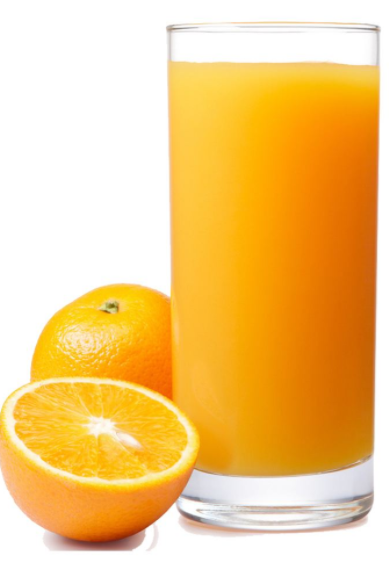

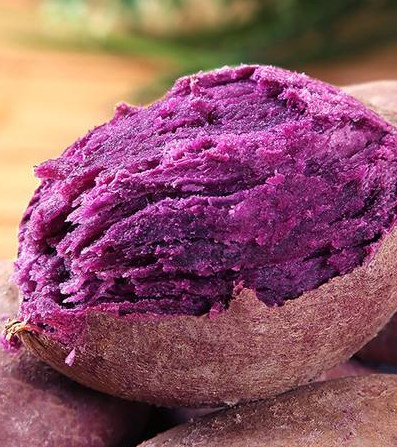

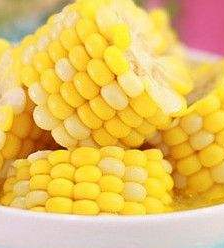

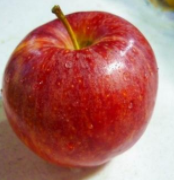

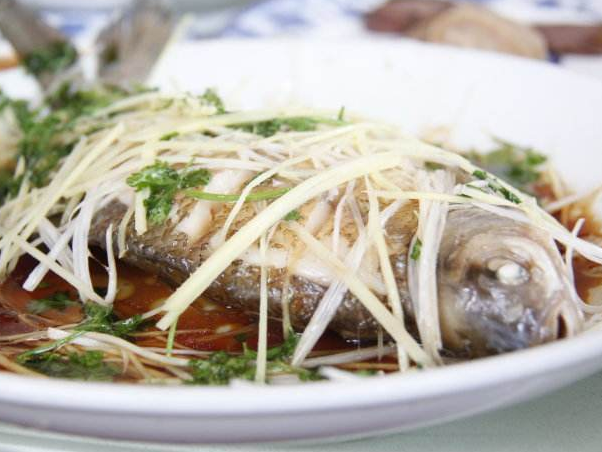


**Fig.1**

Table 1: An example of energy intake over one day for a participant.

Supplement: Supplementary file 1 [file Table_1.docx]
