# Supplementary material for: Effect of a Combined Exercise and Dietary Intervention on Self-Control in Obese Adolescents
Source: Front Psychol. 2019 Jun 28;10:1385. doi: 10.3389/fpsyg.2019.01385 (PMC6610291; doi:10.3389/fpsyg.2019.01385)
Supplement: Supplementary file 2 [file Table_2.docx]

**Supplementary materials**

Table 2: An example of a week in the training program.

|  | Monday | Tuesday | Wednesday | Thursday | Friday | Saturday | Sunday |
| --- | --- | --- | --- | --- | --- | --- | --- |
| 8:00-9:30 | Yoga | Resistance training | Rope skipping | Our door training | Yoga | Aerobic treadmill | Rest |
| 10:00-11:30 | Aerobic treadmill | Aerobic dancing | Static stretching |  | Resistance training | Static stretching |  |
| 15:00-17:00 | Basketball games | Badminton games | Yoga |  | Swimming | Football games |  |
